# Does paternal immunocompetence affect offspring vulnerability to maternal androgens? A study in domestic chickens

**DOI:** 10.1242/bio.045096

**Published:** 2019-11-20

**Authors:** Asmoro Lelono, Diana A. Robledo-Ruiz, Tom V. L. Berghof, Henk K. Parmentier, Bernd Riedstra, Ton G. Groothuis

**Affiliations:** 1 Behavioural Biology, Groningen Institute for Evolutionary Life Sciences, University of Groningen, Nijenborgh 7, 9747 AG, Groningen, The Netherlands; 2 Department of Biology, Faculty of Mathematics and Natural Sciences, University of Jember, 68121 Jember, East Java, The Republic of Indonesia; 3 Department of Animal Sciences, Wageningen University & Research Animal Breeding and Genomics, P.O. Box 338, 6700 AH Wageningen, The Netherlands; 4 Department of Animal Sciences, Wageningen University & Research Adaptation Physiology, P.O. Box 338, 6700 AH Wageningen, The Netherlands

**Keywords:** Immunocompetence, Costs of testosterone, Maternal androgens, Natural antibody

## Abstract

Exposure of yolk androgens can positively stimulate chick growth and competitive ability, but may negatively affect immunity. It has been hypothesized that only chicks from immunologically superior fathers can bear the cost of prenatal exposure to high androgen levels. To test this hypothesis, we paired roosters from two selection lines, one up- and one down-selected for natural antibodies (NAbs), with hens from a control line. We measured yolk testosterone and androstenedione levels, and we injected the treatment group of eggs of each female with testosterone suspended in sesame oil and the control group with sesame oil only. We then measured hatching success and growth, and characterized the humoral and cellular immune responses using three different challenges: a phyto-hemagglutinin, a lipopolysaccharide and a sheep red blood cell challenge. We found that the hatching success, body mass, initial levels of natural antibodies and the chicks’ immunological responses to the three different challenges and development were affected neither by paternal immunocompetence nor by treatment. These results do not support the hypothesis that chicks from low-NAb line fathers are more sensitive to testosterone exposure during embryonic development than chicks from high-NAb line fathers.

## Abstract (precis)

**Summary:** Our study shows that there were no effects of paternal natural antibody line, increased embryonic testosterone exposure or the interaction of both on immunocompetence of chicks.

## INTRODUCTION

Maternal effects are those in which the phenotype of the mother affect the phenotypes of the offspring. This form of non-genetic inheritance can provide mothers with an important tool to adjust the offspring phenotype to the prevailing environmental conditions in which the offspring must survive (Groothuis et al., 2005a). In a variable environment, this is a much more flexible tool for adjustment than genetic inheritance, and of great relevance for understanding evolution, adaptation and the results of breeding programs (Groothuis et al., 2005a; Mousseau and Fox, 1998).

Bird eggs contain considerable amounts of hormones that are deposited in the yolk by the mothers (Anderson and Navara, 2011; Groothuis et al., 2005a; Schwabl, 1996; Von Engelhardt and Groothuis, 2011). Of these hormones, androgens have received the most attention. Several studies have been performed to investigate the effects of prenatal exposure to androgens on the offspring by injecting freshly laid eggs with testosterone (T), androstenedione (A4) (the precursor of the former, but with low affinity to androgen receptors) or both. In summary, increased levels of androgens may induce a variety of beneficial effects for the chicks, such as shorter incubation time (Eising et al., 2001, 2003), faster post-hatching growth (Eising et al., 2001; Groothuis et al., 2005b; Navara et al., 2006; Schwabl, 1996), increased competitive/aggressive behaviors (Eising et al., 2003; Müller et al., 2007; Riedstra et al., 2013; Schwabl, 1996) and increased chances of survival (Eising et al., 2003; Groothuis et al., 2005b; Müller et al., 2007; Von Engelhardt et al., 2006). However, there is substantial variation in both androgen deposition in eggs as well as the results of these egg injection experiments (for a review see Von Engelhardt and Groothuis, 2011). This variation suggests that the beneficial effects may, depending on the context, be constrained by increased costs for the developing embryos. These costs may lie in the detrimental impact that androgens may have on the immune system (Duffy et al., 2000; Folstad and Karter, 1992; Gil et al., 1999; Groothuis et al., 2005b; Müller et al., 2005; Owen-Ashley et al., 2004), which may induce females to strategically vary the allocation of these hormones according to specific environmental variables.

It has also been suggested that the genetic quality of the offspring may determine their vulnerability to the adverse effects of yolk androgens, and therefore, only the offspring of fathers with a genetically based good immune defense would be able to cope with the immune costs of exposure to elevated androgen levels (Gil et al., 1999). As a consequence, females may adjust hormone levels according to the genetic quality of their mate. Indeed, there is evidence suggesting that females differentially allocate androgens according to the expression of the sexual characteristics of their mate, which are considered to be an honest signal of male genetic quality (Andersson and Iwasa, 1996), which has been linked to genetic variation for immunity (Hamilton and Zuk, 1982). For example, it has been shown that mothers produce eggs with higher levels of androgens when paired with highly ornamented males, e.g. attractive color, (Gil et al., 1999), complex song (Gil et al., 2006), larger tail (Gil et al., 2006) and eyespot density (Loyau et al., 2007).

This study aimed to test the hypothesis that exposure to elevated yolk androgen levels would benefit chicks sired by a high-natural-antibody (NAb) line father, but would be detrimental to chicks sired by a low-NAb line father. We therefore paired white leghorn females (*Gallus gallus domesticus*) from a control line with a rooster from either an artificial upward- or downward-selected line for NAbs (Berghof et al., 2018a). NAbs are an essential humoral component of the immune system that provide protection from infection (Ochsenbein et al., 1999). High levels of NAbs in laying chickens were associated with reduced mortality (Star et al., 2007; Sun et al., 2011; Wondmeneh et al., 2015). The chicken lines used in this study had significantly different resistances to avian pathogenic *Escherichia coli* (APEC) from a young age when the immunological challenges were applied: chickens selected for high NAb had two to three times lower mortality and reduced morbidity scores compared to chickens selected for low NAb (Berghof et al., 2019). In addition, the high-NAb line has several indications of improved humoral immunity compared to the low-NAb line, which suggests an improved general (bacterial) immunity (Berghof, 2018; Berghof et al., 2018a). To test our hypothesis on the immunodependent effect of androgen on the development on chicks, we measured the naturally occurring androgen levels in freshly laid eggs. We conducted this test of the effect of mate quality on yolk T deposition for two reasons: (i) to test whether or not mate-dependent differential deposition would occur, as it could have confounded our next experiment and (ii) to test whether females can perceive differences in the male NAb line and, if so, whether they allocate more T to eggs when sired by males of the high-NAb line. We then manipulated yolk T levels of each hen by *in ovo* injections with T suspended in sesame oil in the treatment egg group and with sesame oil only in the control group, resulting in a balanced two-by-two design. We examined embryonic vulnerability to this treatment and examined the effects of treatment and NAb line on cell-mediated immunity by a phyto-hemagglutinin (PHA) challenge and humoral immunity by a sheep red blood cell (SRBC) and lipopolysaccharide (LPS) challenge during the 8 weeks after hatching. In addition, we analyzed the effects of treatment on growth, as this may be traded off for investment in the immune system (Deerenberg et al., 1997; Van der Most et al., 2011; Verhulst et al., 1999). Here, we expected that offspring from T-injected eggs would do better compared to control eggs if they were sired by a male from the high-NAb line, and do worse than controls when sired by the low-NAb line.

## RESULTS

### Yolk T and A4 concentrations

Females did not differently deposit T or A4 in the yolk depending on male line (mean T-level: low NAb line, 11.09±1.01 pg/mg; high NAb line, 10.84±0.67 pg/mg; *t*-test: T=−0.201, d.f.=10, *P*=0.844; mean A4-level: low NAb line, 132.85±12.00 pg/mg; high NAb line, 123.90±5.55 pg/mg; *t*-test, T=−0.656, d.f.=10, *P*=0.526).

### Hatching success and chick growth

During early embryonic development (incubation from day 0 to day 7) 59% of the 223 injected eggs showed proper development (see Table 1). After removal of eggs with no or improper development, 53% of the 132 remaining eggs hatched. There were no differences between T-treated eggs and control eggs in the proportion showing proper development between hens paired with a high- or low-NAb line roosters in the period from 0–7 days of incubation (two-sample *t*-test, T=0.17, d.f.=10, *P*=0.866), from 7 days to hatching (two-sample *t*-test, T=−0.74, d.f.=10, *P*=0.477), nor in overall hatching success (two-sample *t*-test, T=−0.36, d.f.=10, *P*=0.724).

**Table 1.**
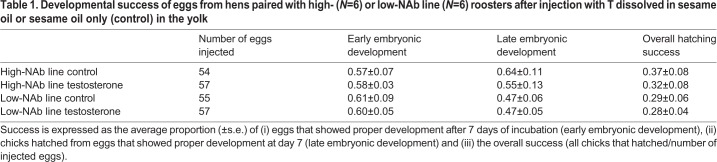
**Developmental success of eggs from hens paired with high- (*N*=6) or low-NAb line (*N*=6) roosters after injection with T dissolved in sesame oil or sesame oil only (control) in the yolk**

At the day of hatching, there were no differences in body mass between T-treated and control chicks, nor was there an effect of paternal line on the differences between T-treated and control chicks and this was also the case at the end of the experiment when chicks were 8 weeks old (see Table 2).

**Table 2.**
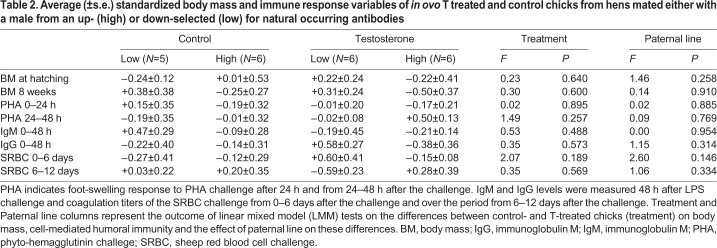
**Average (±s.e.) standardized body mass and immune response variables of *in ovo* T treated and control chicks from hens mated either with a male from an up- (high) or down-selected (low) for natural occurring antibodies**

### Immunocompetence of chicks

#### PHA skin test

All animals had an increased swelling over the first 24 h after the PHA challenge. The average increase was 1.06±0.14 mm and deviated significantly from 0 (one-sample *t*-test: *N*=45, T=7.45, *P*<0.001). The majority (27) of animals showed a decrease in swelling from 24–48 h after injection; 18 did not show a decrease. The average decrease was 0.29±0.15 mm. There was, however, no effect of paternal line on the difference in the response after 24 h between T-treated and control chicks, nor of treatment itself and this was also the case for the response in the period 24–48 h after the challenge (see Table 2).

#### LPS challenge

Just prior to the LPS challenge, the average initial immunoglobulin G (IgG) and immunoglobulin M (IgM) levels were 0.2±0.07 and 2.3±0.10 units, respectively. There was no effect of paternal line on the difference in initial IgG (ANOVA: *F*=0.24, *P*=0.635) or initial IgM levels (*F*=1.01, *P*=0.338) between T-treated and control chicks. There was also no effect of treatment on these levels (IgG: *F*=3.20, *P*=0.104; IgM: *F*=1.99, *P*=0.189). At 48 h after the LPS challenge, all animals had increased IgG and IgM levels. The average increase in IgG level was 0.85±0.06 units, and the average increase in IgM was 0.59±0.05 units. There was no effect of treatment nor paternal line on the difference in the IgG or IgM response after 48 h (see Table 2).

#### SRBC challenge

Out of 44 animals, 38 showed an increase in agglutination titer from day 0 to day 6 after the challenge. The mean increase was 3.23±0.38 units and deviated significantly from 0 (one-sample *t*-test: T=8.47, *P*<0.001). Thirteen of the 44 animals did not show a decrease in coagulation titer from day 6 to day 12. The mean decrease was 1.22±0.27 units, which deviated significantly from 0 (one-sample *t*-test, T=−4.51, *P*<0.001). There was no difference in the response between control and T-treated chicks and there was no effect of paternal line on the difference in response to SRBC over both periods (0–6 days and 6–12 days after the challenge; see Table 2).

## DISCUSSION

We used two chicken lines divergently selected for NAb line with known immunological differences in antibody levels, antibody response, SpAb dynamics and SpAb affinity, and APEC-resistance (Berghof, 2018; Berghof et al., 2018a, 2019). This study aimed to test the hypothesis that exposure to high-androgen yolk content will benefit chicks sired by immunologically high-NAb line males, but it will harm chicks sired by males of low-NAb line. This hypothesis was based on the idea that genetic qualities of offspring can determine their vulnerability to the adverse effects of yolk androgens, and therefore, only the offspring of fathers with good genetic defenses will be able to overcome the costs of decreased immunity due to exposure to high androgen levels (Gil et al., 1999).

### Differential hormone deposition

In several species, it has been shown that females allocate different amounts of androgens to their eggs according to the attractiveness and or quality of their mate (Garcia-Fernandez et al., 2010; Gil et al., 1999; Gilbert et al., 2006). These hormones can have beneficial effects for chicks (Groothuis et al., 2005b), which raises the question of why avian mothers do not provide all of their eggs with ample amounts of androgens. We tested the hypothesis that, given that exposure to elevated amounts of androgens is harmful to the immune system (see Introduction), only chicks sired by high-NAb line fathers can bear the costs bestowed upon them when embryonic development takes place in androgen-rich environments (Gil et al., 1999).

In this study, we firstly tested whether female laying hens also differentially deposited yolk androgens according to mate NAb line as measured in immunity. The immunity is relevant because the benefits of being exposed to androgens during early development may be traded off against the potential costs inflicted on the immunocompetence – the potential cost being a heritable trait (Berghof et al., 2015, 2018b) – of offspring by this early exposure (Gil et al., 1999; Groothuis et al., 2005b; Von Engelhardt and Groothuis, 2011). We then paired up hens from a control line with males from a line up-selected and a line down-selected for total NAb levels (Berghof et al., 2018a) and collected the eggs they produced.

We found that eggs collected from females paired with these two types of males did not differ in the concentrations of the two most common androgens found in avian eggs: A4 and T. This lack of a difference may have several causes. Firstly, the resulting deposition could have been masked by a mechanism in which hens further differentiated their hormone allocation according to offspring sex. Such a mechanism is present in the ancestral species of laying hens, the red junglefowl (Lelono et al., 2019), in laying hens themselves (Müller et al., 2002), house sparrows (Badyaev et al., 2005) and has also been demonstrated in other species e.g. zebra finches (Gilbert et al., 2005; Rutstein et al., 2005). This may make sense as one could expect that females invest more in sons relative to daughters when paired with high-NAb line males in the harem system of fowl and more in daughters when paired with low-NAb line males (Clutton-Brock, 1989). Unfortunately, we could not determine the sex of the embryos at the day of oviposition. Secondly, our sexually naive females might have been unable to assess whether there were physical differences between males of the two lines. In our experiment, females were randomly paired with one type of male, without awareness of the other type. Females could therefore not have assessed the relative NAb line of one type of male over the other.

Moreover, the males did not differ in body mass and as the combs were dubbed at an early age, to prevent damage in the housing system they were kept in before arriving at our facility; there were also no noticeable differences in comb size. Thirdly, phenotypic and genetic differences in immunocompetence of the males after four generations of divergent selection for NAb might not have been large enough for females to distinguish the level of the male NAb concentration. Therefore, the lack of evidence for the influence of male immunological quality on differential yolk hormone deposition by females does not jeopardize our experimental design but facilitates the injection approach, as the experimental elevation of T was not confounded by already existing hormone differences between the experimental groups.

### Immunocompetence and embryonic T exposure

In this study, we tested whether the genetically based immunocompetence of roosters determined offspring vulnerability to the potential adverse effects of yolk androgens, with the main focus on differences in immunocompetence early in life. With our immunological challenges, we tested both arms of the immune system, which differed in how energetically demanding they were (Kankova et al., 2018). Although it is clear that all three challenges provoked an immune response in the majority of chicks, we found no evidence that the immunocompetence of chicks sired by the low-NAb line roosters was compromised more by our treatment than that of chicks sired by the high-NAb line roosters during the first weeks of life.

The hypothesis could be rescued by three possibilities. Firstly, females compensated for low parental NAb line by adjusting other components of the eggs such as depositing immune enhancing factors like carotenoids, which we did not measure. However, this explanation would assume that the hens could distinguish male NAb line differences for which we have no evidence (see above). Another possibility is that the effects of embryonic exposure may have only long-lasting consequences. The immune system of chicks in the period that we challenged them was still developing (Apanius, 1998), therefore the effects of embryonic T exposure may have been only small and difficult to detect. However, the immune-modulating effect of yolk T has repeatedly been found in the chick stage in which we did our tests (Duffy et al., 2000; Folstad and Karter, 1992; Gil et al., 1999; Groothuis et al., 2005b; Kankova et al., 2018; Müller et al., 2005; Owen-Ashley et al., 2004), albeit in an inconsistent manner that might depend on the testing age, dosage or species (also briefly reviewed in Kankova et al., 2018) a study that tested the effect of selection on yolk T and T-injection *in ovo* in a similar design and using partly the same tests as we did). In addition, male genetic effects on immune competence are halved in the offspring, which may also account for the lack of significant differences. Finally, elevated prenatal exposure to yolk T may have effects that we did not test. For example, it has been found that yolk T increases metabolic rate (Tobler et al., 2007). Since metabolic rate determines the rate of almost all biological activities (Brown et al., 2004), and especially parts of the immune system (e.g. Kankova et al., 2018), it may be possible that under natural conditions where food is not available *ad libitum*, detrimental effects either in the chick phase or in a later stage may be more pronounced. However, the original hypothesis, that for immune defense, the high-NAb line father is the reason for mate quality-dependent maternal hormone deposition, cannot be supported by our study.

### Hatching, growth and its relation to NAb line

We did not find any indications that early exposure to T affected hatching success differently for the two lines; not during early embryonic development and not during late embryonic development. Since immunological differences between the paternal lines have a heritable component (Berghof et al., 2015, 2018b) and growth may trade off against immunological quality (Deerenberg et al., 1997; van der Most et al., 2011; Verhulst et al., 1999), we expected that the effects of early T exposure would affect growth in the two lines differentially. This was not the case. There was no effect of paternal line on differences in body mass between T-treated and control siblings both at the time of hatching and at the age of 8 weeks, which also indicated that the benefits of developing in androgen-rich environments was not dependent on the immunocompetence of the paternal line.

In contrast to our observation and to the proposed hypothesis of trade-off, body weight differences were actually observed in the NAb selection experiment: higher body weights between 0 weeks of age (i.e. hatch) and approximately 30 weeks of age were found for chickens selected for high-NAb levels compared to chickens selected for low-NAb levels (Berghof, 2018). A possible lack of significant differences could be due to a lack of statistical power or a lack of social competition for food (in the NAb selection lines, chickens were housed with 80–100 individuals per pen). However, more likely were the maternal environmental effects between 0 and 8 weeks of age (0 weeks: 56%, 4 weeks: 7%, 8 weeks: 3% variation explained by maternal effects; Berghof et al. in prep.). Since the females were unselected for NAb levels (and thus indirectly for body mass differences) and in combination with only half of the paternal genetic effect, the differences in body mass (and NAb, as mentioned above) were too small to detect. Regardless, we also found no effects of treatment itself on body mass. One possibility that may have confounded this lack of effect is that there was a mechanism in action, as mentioned above, in which hens differentially allocated hormones to eggs in an offspring sex-dependent way. Fowl are capable of offspring-sex-dependent hormone deposition (Lelono et al., 2019; Müller et al., 2002).

### In conclusion

Our study found no support for the hypothesis that only offspring of high-NAb line fathers, reflected by their paternal immunocompetence, can bear the cost that increased embryonic exposure to T has on their immune system. We found no effects of the interaction between paternal immunocompetence represented by NAb levels and *in ovo* T treatment on hatching success, body mass or immunocompetence (both cellular as well as humoral) of their chicks. However, it is also possible that the principle component affected by early embryonic exposure to T is not the immunity directly, but metabolism, which may only have negative consequences in the long term for immunity and positive effects for growth.

## MATERIALS AND METHODS

### Ethics

All experimental procedures were carried out according to the regulation of Dutch law for laboratory animals and approved by the animal experimentation committee of the University of Groningen the Netherlands (license DEC 6710B Amendment 003 in 2015).

All handling and treatment of animals were carried out by animal caretakers and experienced scientists with a license [certificate number 685412, DG VGZ/VVP (Stcrt.135), 25 January 2013].

### Animals and housing

We used six white leghorn (*Gallus gallus domesticus*) roosters from two NAb selection lines from a breeding stock of the Wageningen University & Research, The Netherlands. These lines were divergently selected for total [i.e. heavy and light chain-binding ELISA recognizing IgM, immunoglobulin A (IgA) and IgG isotypes] keyhole-limpet hemocyanin-binding NAb titers at 16 weeks of age (Berghof et al., 2018a). The roosters used differed substantially in total NAb levels at the age of 16 weeks [mean±s.e. IgM: high-NAb line=5.58±0.19 (*N*=6); low-NAb line=2.76±1.12 (*N*=6); *t*-test: T=5.66, d.f.=10, *P*<0.001; mean IgG (±s.e.): high-NAb line=7.03±0.14, low-NAb line=3.56±0.59; *t*-test: T=10.02, d.f.=10, *P*<0.001]. Male body mass did not differ between the two lines (high-NAb line=1481.2±41.65 g, low NAb line=1388.8±42.85 g; *t*-test: T=1.54, d.f.=10, *P*=0.15). Furthermore, we obtained 12 white females of the unselected WA control line from Hendrix Genetics, Boxmeer, The Netherlands. This line also served as the base population for the NAb selection lines.

We then paired six females with six roosters from the high-NAb line, and six females with six roosters from the low-NAb line. These 12 pairs were housed individually in pens of 1.5×4×2 m (w×l×h), visually isolated from other pairs, under a natural light regime. Every pen contained a perch, a sand area for dust bathing and a nesting site. Food (laying pellets, Kasper Fauna food, article number 601820) and water were provided *ad libitum*. Additionally, hens received a handful of mixed grains (Kasper Fauna food, article number 384020) once a week.

### Design and egg collection

From 6 days after housing onward, we first collected four eggs per hen (total=48 eggs) to determine hormone levels. These eggs were marked and frozen at −20°C awaiting hormone analyses. Four weeks later, we collected 18–19 eggs of each hen (total 223 eggs) that were treated with T or sham-treated (see below). Eggs were placed in an artificial incubator at 37.5°C with 60% humidity. Turning of the eggs took place until day 20 when all the eggs were placed in separate box to avoid mixing with other chicks. We then also increase humidity from 60% up to 80% before hatching. We denoted line, treatment and maternal origin of eggs that did not hatch.

### Yolk hormone analyses

Yolk T and A4 concentrations were quantified by radioimmunoassay after extraction of hormones. To extract the hormones, 220 mg of yolk/milliQ water mixture (1+1) was weighed (accuracy 1 mg), 300 µl of milliQ water was added and 50 µl of 3H-labeled T (NET553, Perkin Elmer) was added to trace the recovery of extracted hormones during the extraction procedure. This solution was incubated for 15 min at 37°C before being extracted in 2 ml of diethyl-ether/petroleum-ether (DEE/PE, 70/30 v/v) by vortexing for 60 s. Extracted samples were centrifuged at 2000 rpm for 3 min (4°C) to separate the ether phase, the samples were snap-frozen and the ether/hormone phase decanted into a fresh tube. The extraction procedure was repeated twice with an additional 2 ml of DEE/PB, vortexed for 30 s and 15 s, respectively. Next, the extracts were dried under nitrogen at 37°C. Hormone extracts were rinsed in 2 ml of 70% methanol to precipitate any lipids and stored overnight at −20°C. Subsequently, the tubes were centrifuged, decanted into a fresh tube, re-dried under nitrogen at 50°C and stored at −20°C. Prior to assay, extracts were thawed and dissolved in 300 µl phosphate-buffered-saline with gelatine. Recoveries of the initially added labeled T were measured in a subsample of this solution using scintillation cocktail (Ultima Gold, Perkin Elmer) and radioactivity counted on a liquid scintillation counter. The average recovery was 86% (s.d. 2.8%). Subsequently, 25 µl of the extracted sample was used for T determination using the same kit as above. Standards were prepared using dilution series from pre-prepared stock and ranged from 0.08–20 ng/ml. For A4 determination 50 µl of the extracted sample (dilution ×21), using a commercial kit (DSL-3800, Beckman Coulter GmbH, Sinsheim, Germany). ‘Pools’ of yolks were used as external controls, and intra assay for T and A4 was 2.53%, and 3.06% respectively.

### Set up of the injection experiment

For each hen, half of the collected eggs were randomly assigned to the control treatment and the other half to the T treatment. Yolk T and control injections were performed, after placing the eggs horizontally for 30 min (allowing the yolk with the blastodisc to float up to the top) using a 0.5 ml insulin syringe. A small hole (∼2 mm) was drilled in the eggshell, and the needle was inserted at 45°C into the yolk, where the substances were slowly injected. Holes were sealed with small drops of melted candle wax. Eggs were either injected with 100 μl vehicle only or 50 ng T (46923, Sigma-Aldrich) and dissolved in 100 μl vehicle (sterile sesame oil), which constituted the amount of ∼2 s.d. of the naturally occurring T levels (mean±s.d.=127.2±23.3 ng/total yolk), which we determined from the first four eggs collected from each hen. This way we obtained four experimental groups: (1) high-control (HC), (2) high-testosterone (HT), (3) low-control (LC) and (4) low-testosterone (LT) (see Table 1 for the number of injected eggs per group). Injected eggs were incubated at 37.5°C with 60% humidity.

### Chick housing

Directly after hatching, chicks were weighed and colored rubber bands for individual recognition were fitted. After 2 weeks, chicks were also fitted by a numbered metal wing tag. From hatching until day 14, chicks were housed together in a circular enclosure (diameter: 1.5 m). Food (Kasper Fauna food, article number 650004) and water was provided *ad libitum*. At 14 days of age, we sexed chicks based on physical characteristics (body mass and the rise of comb) and confirmed the sex with a DNA test (Berghof, 2018). At the same age, 48 chicks were selected from the entire group and housed in six groups of eight (treatment and control of each sex from each mother and each father line) in metal cages (w×l×h: 1×1×1 m). At week 4, the cages were enlarged to 2×1×1 m. Food (Kasper Fauna food, article number 600320) and water were provided *ad libitum*. In all housing conditions, the heat was provided by an incandescent infrared heat lamp 230–250 V BR 125–250 W, and the floor was covered with wood shavings. Body mass was recorded weekly to asses growth from day of hatching until 8 weeks after hatching.

### Cell-mediated immunity: PHA challenge

The first immunological test we performed was the PHA skin test, which is used to measure cell-mediated immunity *in vivo*. PHA stimulates a local swelling caused by the perivascular accumulation of T-lymphocytes and macrophage infiltration (Smits et al., 1999). At day 23, chicks were injected subcutaneously with 0.04 ml of a 5 mg/ml solution of PHA-P (L1668, Sigma-Aldrich) dissolved in PBS into the ball of the left foot (for details see Müller et al., 2003). We measured the PHA response by comparing the change in the average of three repeated measurements (using a sliding caliper) of the height of the ball of the foot (from the base of the hind toe to the top of the foot bowl when placing the foot at 90° to the tarsus) from just prior to injection to that after 24 h and the change from 24 h to 48 h after injection.

### Humoral immunity: LPS challenge

To test the humoral immune response of the chicks, we injected LPS from *E. coli* cell walls (LPS antigen *E.* *coli* O127: B8, Sigma-Aldrich) 32 days after hatching. This LPS challenge mimics an infection caused by gram-negative bacteria and is a potent pathogen-associated molecular pattern that induces the release of inflammatory cytokines, which can be accompanied by the production of antibodies (Müller et al., 2005). In this experiment, we applied 0.1 mg LPS antigen dissolved in 0.1 ml PBS (concentration 1 mg/ml) once by intraperitoneal injection. We took two blood samples by punctuating the brachial vein of the left wing with a 25 G needle: one just before the LPS injection and one 48 h after the injection (Müller et al., 2004). Blood (c. 50 µl) was collected in a vial with the EDTA (9 g/ml) and centrifuged for 10 min at 10.000 rpm. The plasma was then obtained and stored at −20°C until analysis.

Titers of immunoglobulin isotypes IgM, and IgG-binding keyhole limpet hemocyanin (KLH) were determined in individual plasma samples by an indirect two-step ELISA as described by Berghof et al. (2018b). Briefly, flat-bottomed 96-well medium binding plates (Greiner Bio-One, Alphen a/d Rijn, The Netherlands) were coated with 2 μg/ml KLH (Sigma-Aldrich) in 100 μl coating buffer (5.3 g/l Na2CO3 and 4.2 g/l NaHCO3; pH 9.6), and incubated at 4°C overnight. After washing with tap water containing 0.05% Tween 20 for 6 s, plates were tapped dry. Plasma samples were 1:10 pre-diluted (for IgM and IgG analyses) with dilution buffer [phosphate buffered saline (PBS; 10.26 g/l Na2HPO4_H2O, 2.36 g/l KH2PO4 and 4.50 g/l NaCl; pH 7.2) containing 0.5% normal horse serum and 0.05% Tween 20]. Pre-dilutions were stored at 4°C for the next day or were frozen until use. Pre-dilutions were diluted with dilution buffer. Tested plasma dilutions were 1:160, 1:640, 1:2560 and 1:10,240. Duplicate standard positive plasma samples (a pool of approximately half of the males of the NAb base population) were stepwise diluted with dilution buffer. The plates were incubated for 1.5 h at 23°C. After washing, plates were incubated with 1:20,000-diluted goat-anti-chicken IgM heavy chain labeled with horse radish peroxidase (HRP) (cat# A30-102P, RRID:AB_66857), or 1:40,000-diluted goat-anti-chicken IgG(Fc) labeled with HRP (cat# A30- 104P, RRID:AB_66843) (all polyclonal antibodies from Bethyl Laboratories, Montgomery, TX, USA) and incubated for 1.5 h at 23°C.

After washing, binding of the antibodies to KLH was visualized by adding 100 μl substrate buffer [containing reverse osmosis purified water, 10% tetramethylbenzidine buffer (15.0 g/l sodium acetate and 1.43 g/l urea hydrogen peroxide; pH 5.5), and 1% tetramethylbenzidine (8 g/l TMB in DMSO)] at room temperature. After 15 min, the reaction was stopped with 50 μl of 1.25 M H_2_SO_4_. Extinctions were measured with a Multiskan Go (Thermo Fisher Scientific) at 450 nm. Antibody titers were calculated based on log2 values of the dilutions that gave extinction closest to 50% of EMAX, where EMAX represents the mean of the highest extinction of the standard positive plasma samples, thereby partly correcting for plate differences (Berghof et al., 2018b).

### Humoral immunity: SRBC challenge

SRBC challenges are commonly used to determine the response of the avian humoral immune system (Müller et al., 2004). We used SRBC (Glutaraldehyde Stabilized/product no. R3378, Sigma-Aldrich) 45 days after hatching to investigate long-term consequences of embryonic androgen exposure. Shortly before immunization, a blood sample (200 µl) was taken to measure background antibody concentration. Chicks were then intraperitoneally injected with 500 µl of a 2% SRBC suspension dissolved in PBS. Six and 12 days post-immunization, birds were re-sampled. Blood (c. 50 µl) was collected in a vial with the EDTA (9 g/ml) and centrifuged for 10 min at 10,000 rpm. Plasma was separated from the blood cell and stored at −20°C until further analysis.

The quantification of the immune response induced by the SRBC challenge was performed following the protocol for haemagglutination described by (Ros et al., 1997). The titers were scored visually by an experienced person (B.R.). The highest dilution at which the SRBC still agglutinated, which indicated the amount of antibodies in the samples, was recorded. The measures are represented as integers on a log scale, and the mean value of the two replicates of each sample was used to calculate the initial and final antibody concentration.

### Statistical analyses

To test whether females mated with roosters of the high-NAb line deposited more androgens in their eggs than females mated with roosters of the low-NAb line, we used two-sample *t*-tests on the average hormone deposition per female. To test the effect of (the vulnerability to) T on successful embryonic development in eggs produced by hens mated with high- or low-NAb line roosters, we used two-sample *t*-tests, (using the proportion of successfully developed control eggs minus the proportion successfully developed T-injected eggs for each hen) in each condition. The possible effects were tested for three different periods: (i) during early embryonic development we calculated the proportion of eggs showing proper development after 7 days of incubation, (ii) during late embryonic development we calculated the proportion of chicks that eventually hatched from eggs showing proper development after 7 days of incubation, and (iii) over the total period of incubation we calculated the hatching success (number of chicks hatched/total number of eggs injected).

To test the hypothesis that only high-NAb line fathers produce offspring that can bear the cost of increased T levels, to avoid pseudo replication and reduce the number of predictor variables, we first standardized body mass and immune response variables (Z-transformation) per sex. We then averaged the standardized values of all siblings from each mother and calculated the difference between T-treated and control chicks in these variables. Previously, we had run the linear mixed model (LMM), and the range of the *P*-value was 0.216 up to 0.866. Subsequently, we tested the effect of line and the effect of treatment (deviation from the intercept) on the differences in body mass at hatching and at week 8 in a multivariate ANOVA. The differences in immune variables between T- and control-treated animals within females were tested using LMMs with the standardized difference in body mass at the start of the challenge as a covariate. An identical approach was taken to test the effects of line and treatment on the initial IgG and IgM levels (just prior to the challenge) and maternal identity as random effect. Mean are presented with the standard error of the mean. One hen mated with a male of the low-NAb line did not produce chicks from control-injected eggs.

All statistical analyses were performed with SPSS23.

## References

[BIO045096C1] Anderson E. M. and Navara K. J. (2011). Steroid hormone content of seminal plasma influences fertilizing ability of sperm in White Leghorns. *Poult. Sci.* 90, 2035-2040. 10.3382/ps.2010-01299 21844270

[BIO045096C2] Andersson M. and Iwasa Y. (1996). Sexual selection. *Trends Ecol. Evol.* 11, 53-58. 10.1016/0169-5347(96)81042-1 21237761

[BIO045096C3] Apanius V. (1998). Stress and immune defense. *Adv. Study Behav.* 27, 133-153. 10.1016/S0065-3454(08)60363-0

[BIO045096C4] Badyaev A. V., Schwabl H., Young R. L., Duckworth R. A., Navara K. J. and Parlow A. F. (2005). Adaptive sex differences in growth of pre-ovulation oocytes in a passerine bird. *Proc. R. Soc. B Biol. Sci.* 272, 2165-2172. 10.1098/rspb.2005.3194 PMC1559945 16188605

[BIO045096C5] Berghof T. V. L. (2018). Selective breeding on natural antibodies in chickens. *PhD Dissertation*. Wageningen, Wageningen University ISBN 9789463437257 - 199.

[BIO045096C6] Berghof T. V. L., van Der Klein S. A. S., Arts J. A. J., Parmentier H. K., van der Poel J. J. and Bovenhuis H. (2015). Genetic and non-genetic inheritance of natural antibodies binding keyhole limpet hemocyanin in a purebred layer chicken line. *PLoS ONE* 10, e0131088 10.1371/journal.pone.0131088 26114750 PMC4482680

[BIO045096C7] Berghof T. V. L., Arts J. A. J., Bovenhuis H., Lammers A., van der Poel J. J. and Parmentier H. K. (2018a). Antigen-dependent effects of divergent selective breeding based on natural antibodies on specific humoral immune responses in chickens. *Vaccine* 36, 1444-1452. 10.1016/j.vaccine.2018.01.063 29409681

[BIO045096C8] Berghof T. V. L., Visker M. H. P. W., Arts J. A. J., Parmentier H. K., van der Poel J. J., Vereijken A. L. J. and Bovenhuis H. (2018b). Genomic region containing toll-like receptor genes has a major impact on total IGM antibodies including KLH-binding IGM natural antibodies in chickens. *Front. Immunol.* 8, 1879 10.3389/fimmu.2017.01879 29375555 PMC5767321

[BIO045096C9] Berghof T. V. L., Matthijs M. G. R., Arts J. A. J., Bovenhuis H., Dwars R. M., van der Poel J. J., Visker M. H. P. W. and Parmentier H. K. (2019). Selective breeding for high natural antibody level increases resistance to avian pathogenic Escherichia coli (APEC) in chickens. *Dev. Comp. Immunol.* 93, 45-57. 10.1016/j.dci.2018.12.007 30579935

[BIO045096C10] Brown J. H., Gillooly J. F., Allen A. P., Savage V. M. and West G. B. (2004). Toward a metabolic theory of ecology. *Ecology* 85, 1771-1789. 10.1890/03-9000

[BIO045096C11] Clutton-Brock T. H. (1989). Review lecture: mammalian mating systems. *Proc. R. Soc. B Biol. Sci.* 236, 339-372. 10.1098/rspb.1989.0027 2567517

[BIO045096C12] Deerenberg C., Arpanius V., Daan S. and Bos N. (1997). Reproductive effort decreases antibody responsiveness. *Proc. R. Soc. Lond. B* 264, 1021-1029. 10.1098/rspb.1997.0141

[BIO045096C13] Duffy D. L., Bentley G. E., Drazen D. L. and Ball G. F. (2000). Effects of testosterone on cell-mediated and humoral immunity in non-breeding adult European starlings. *Behav. Ecol.* 11, 654-662. 10.1093/beheco/11.6.654

[BIO045096C14] Eising C. M., Eikenaar C., Schwabl H. and Groothuis T. G. G. (2001). Maternal androgens in black-headed gull (Larus ridibundus) eggs: consequences for chick development. *Proc. R. Soc. Lond. B* 268, 839-846. 10.1098/rspb.2001.1594 PMC1088678 11345330

[BIO045096C15] Eising C. M., Müller W., Dijkstra C. and Groothuis T. G. G. (2003). Maternal androgens in egg yolks: Relation with sex, incubation time and embryonic growth. *Gen. Comp. Endocrinol.* 132, 241-247. 10.1016/S0016-6480(03)00090-X 12812771

[BIO045096C16] Folstad I. and Karter A. J. (1992). Parasites, bright males, and the immunocompetence handicap. *Am. Nat.* 139, 603-622. 10.1086/285346

[BIO045096C17] Garcia-Fernandez V., Guasco B., Tanvez A., Lacroix A., Cucco M., Leboucher G. and Malacarne G. (2010). Influence of mating preferences on yolk testosterone in the grey partridge. *Anim. Behav.* 80, 45-49. 10.1016/j.anbehav.2010.03.023

[BIO045096C18] Gil D., Graves J. A., Hazon N. and Wells A. (1999). Male attractiveness and differential testosterone investment in zebra finch eggs. *Science* 286, 126-128. 10.1126/science.286.5437.126 10506561

[BIO045096C19] Gil D., Ninni P., Lacroix A., De Lope F., Tirard C., Marzal A. and Pape Møller A. (2006). Yolk androgens in the barn swallow (Hirundo rustica): a test of some adaptive hypotheses. *J. Evol. Biol.* 19, 123-131. 10.1111/j.1420-9101.2005.00981.x 16405584

[BIO045096C20] Gilbert L., Rutstein A. N., Hazon N. and Graves J. A. (2005). Sex-biased investment in yolk androgens depends on female quality and laying order in zebra finches (Taeniopygia guttata). *Naturwissenschaften* 92, 178-181. 10.1007/s00114-004-0603-z 15668780

[BIO045096C21] Gilbert L., Williamson K. A., Hazon N. and Graves J. A. (2006). Maternal effects due to male attractiveness affect offspring development in the zebra finch. *Proc. R. Soc. B* 273, 1765-1771. 10.1098/rspb.2006.3520 PMC1634786 16790409

[BIO045096C22] Groothuis T. G. G., Müller W., von Engelhardt N., Carere C. and Eising C. (2005a). Maternal hormones as a tool to adjust offspring phenotype in avian species. *Neurosci. Biobehav. Rev.* 29, 329-352. 10.1016/j.neubiorev.2004.12.002 15811503

[BIO045096C23] Groothuis T. G. G., Eising C. M., Dijkstra C. and Müller W. (2005b). Balancing between costs and benefits of maternal hormone deposition in avian eggs. *Biol. Lett.* 1, 78-81. 10.1098/rsbl.2004.0233 17148133 PMC1629043

[BIO045096C24] Hamilton W. D. and Zuk M. (1982). Heritable true fitness and bright birds: a role for parasites? *Science* 218, 384-387. 10.1126/science.7123238 7123238

[BIO045096C25] Kankova Z., Zeman M., Ledecka D. and Okuliarova M. (2018). Variable effects of elevated egg yolk testosterone on different arms of the immune system in young quail. *Gen. Comp. Endocrinol.* 256, 30-36. 10.1016/j.ygcen.2017.07.021 28736225

[BIO045096C26] Lelono A., Riedstra B. and Groothuis T. G. G. (2019). The relationship between male social status, ejaculate and circulating testosterone concentration and female yolk androgen transfer in red junglefowl (*Gallus gallus*). *Horm. Behav.* 116, 104580 10.1016/j.yhbeh.2019.104580 31472122

[BIO045096C27] Loyau A., Saint Jalme M., Mauget R. and Sorci G. (2007). Male sexual attractiveness affects the investment of maternal resources into the eggs in peafowl (Pavo cristatus). *Behav. Ecol. Sociobiol.* 61, 1043-1052. 10.1007/s00265-006-0337-3

[BIO045096C28] Mousseau T. A. and Fox C. W. (1998). The adaptive significance of maternal effects. *Trends Ecol. Evol.* 13, 403-407. 10.1016/S0169-5347(98)01472-4 21238360

[BIO045096C29] Müller W., Eising C. M., Dijkstra C. and Groothuis T. G. G. (2002). Sex differences in yolk hormones depend on maternal social status in leghorn chickens (*Gallus gallus domesticus*). *Proc. R. Soc. Lond. B* 269, 2249-2255. 10.1098/rspb.2002.2159 PMC1691150 12427318

[BIO045096C30] Müller W., Dijkstra C. and Groothuis T. G. G. (2003). *Inter-se*xual differences in T-cell-mediated immunity of black-headed gull chicks (Larus ridibundus) depend on the hatching order. *Behav. Ecol. Sociobiol.* 55, 80-86. 10.1007/s00265-003-0681-5

[BIO045096C31] Müller W., Groothuis T. G. G., Riedstra B., Dijkstra C., Alatalo V. and Siitari H. (2004). Short- and long-term consequences of prenatal androgen exposure on the immune system of black-headed gulls. PhD dissertation University of Groningen, The Netherlands, 129-146. (https://www.rug.nl/research/portal/publications/maternal-phenotypic-engineering(4515ee41-6505-4732-a152-1e82f6c779f0).html).

[BIO045096C32] Müller W., Groothuis T. G. G., Kasprzik A., Dijkstra C., Alatalo R. V. and Siitari H. (2005). Prenatal androgen exposure modulates cellular and humoral immune function of black-headed gull chicks. *Proc. R. Soc. B* 272, 1971-1977. 10.1098/rspb.2005.3178 PMC1559883 16191605

[BIO045096C33] Müller W., Deptuch K., López-Rull I. and Gil D. (2007). Elevated yolk androgen levels benefit offspring development in a between-clutch context. *Behav. Ecol.* 18, 929-936. 10.1093/beheco/arm060

[BIO045096C34] Navara K. J., Hill G. E. and Mendonça M. T. (2006). Yolk androgen deposition as a compensatory strategy. *Behav. Ecol. Sociobiol.* 60, 392-398. 10.1007/s00265-006-0177-1

[BIO045096C35] Ochsenbein A. F., Fehr T., Lutz C., Suter M., Brombacher F., Hengartner H. and Zinkernagel R. M. (1999). Control of early viral and bacterial distribution and disease by natural antibodies. *Science* 286, 2156-2159. 10.1126/science.286.5447.2156 10591647

[BIO045096C36] Owen-Ashley N. T., Hasselquist D. and Wingfield J. C. (2004). Androgens and the immunocompetence handicap hypothesis: unraveling direct and indirect pathways of immunosuppression in song sparrows. *Am. Nat.* 164, 490-505. 10.1086/423714 15459880

[BIO045096C37] Riedstra B., Pfannkuche K. A. and Groothuis T. G. G. (2013). Increased exposure to yolk testosterone has feminizing effects in chickens, Gallus gallus domesticus. *Anim. Behav.* 85, 701-708. 10.1016/j.anbehav.2013.01.011

[BIO045096C38] Ros A. F. H., Groothuis T. G. G. and Apanius V. (1997). The relation among gonadal steroids, immunocompetence, body mass, and behavior in young black-headed gulls (*Larus Ridibundus*). *Am. Nat.* 150, 201-219. 10.1086/286063 18811282

[BIO045096C39] Rutstein A. N., Gorman H. E., Arnold K. E., Gilbert L., Orr K. J., Adam A., Nager R. and Graves J. A. (2005). Sex allocation in response to paternal attractiveness in the zebra finch. *Behav. Ecol.* 16, 763-769. 10.1093/beheco/ari052

[BIO045096C40] Schwabl H. (1996). Maternal testosterone in the avian egg enhances postnatal growth. *Comp. Biochem. Physiol. A Physiol.* 114, 271-276. 10.1016/0300-9629(96)00009-6 8759148

[BIO045096C41] Smits J. E., Bortolotti G. R. and Tella J. L. (1999). Simplifying the phytohaemagglutinin skin-testing technique in studies of avian immunocompetence. *Funct. Ecol.* 13, 567-572. 10.1046/j.1365-2435.1999.00338.x

[BIO045096C42] Star L., Frankena K., Kemp B., Nieuwland M. G. B. and Parmentier H. K. (2007). Natural humoral immune competence and survival in layers. *Poult. Sci.* 86, 1090-1099. 10.1093/ps/86.9.1894 17495078

[BIO045096C43] Sun Y., Parmentier H. K., Frankena K. and van der Poel J. J. (2011). Natural antibody isotypes as predictors of survival in laying hens. *Poult. Sci.* 90, 2263-2274. 10.3382/ps.2011-01613 21934009

[BIO045096C44] Tobler M., Nilsson J.-Å. and Nilsson J. F. (2007). Costly steroids: egg testosterone modulates nestling metabolic rate in the zebra finch. *Biol. Lett.* 3, 408-410. 10.1098/rsbl.2007.0127 17456447 PMC2390662

[BIO045096C45] Van der Most P. J., de Jong B., Parmentier H. K. and Verhulst S. (2011). Trade-off between growth and immune function: a meta-analysis of selection experiment. *Funct. Ecol.* 25, 74-80. 10.1111/j.1365-2435.2010.01800.x

[BIO045096C46] Verhulst S., Dieleman S. J. and Parmentier H. K. (1999). A tradeoff between immunocompetence and sexual ornamentation in domestic fowl. *Proc. Natl. Acad. Sci. USA* 96, 4478-4481. 10.1073/pnas.96.8.4478 10200287 PMC16357

[BIO045096C47] Von Engelhardt N. and Groothuis T. (2011). Maternal hormones in avian eggs. In *Hormones and Reproduction of Vertebrates* (ed. Norris D. and Lopez K. H.), pp. 91-127. Elsevier Ltd.

[BIO045096C48] von Engelhardt N., Carere C., Dijkstra C. and Groothuis T. G. G. (2005). Sex-specific effects of yolk testosterone on survival, begging and growth of zebra finches. *Proc. R. Soc. B Biol. Sci.* 273, 65-70. 10.1098/rspb.2005.3274 PMC1560008 16519236

[BIO045096C49] Wondmeneh E., Van Arendonk J. A. M., Van der Waaij E. H., Ducro B. J. and Parmentier H. K. (2015). High natural antibody titers of indigenous chickens are related with increased hazard in confinement. *Poult. Sci.* 94, 1493-1498. 10.3382/ps/pev107 25910906 PMC4991063

